# Identifying and predicting food parenting practice profiles among Canadian parents

**DOI:** 10.1186/s12966-021-01119-6

**Published:** 2021-05-04

**Authors:** Claire N. Tugault-Lafleur, Olivia De-Jongh González, Teresia M. O’Connor, Sheryl O. Hughes, Louise C. Mâsse

**Affiliations:** 1 Department of Family Relations and Applied Nutrition, The University of Guelph, Guelph, ON N1G 1Y1 Canada; 2 School of Population and Public Health University of British Columbia, BC Children’s Hospital Research Institute, F508-4490 Oak Street, Vancouver, BC V5Z 4H4 Canada; 3 USDA/ARS Children’s Nutrition Research Center, Baylor College of Medicine, 1100 Bates St, Houston, TX 77030 USA

**Keywords:** Food parenting practices, Eating behaviours, Children, Latent class analysis

## Abstract

**Background:**

Food parenting practices (FPP) can affect children’s eating behaviours, yet little is known about how various FPP co-occur. The primary aim was to identify profiles of FPPs use among Canadian parents. Secondary aims included examining sociodemographic correlates of FPP profiles and evaluating whether children’s eating behaviours differed across FPP profiles.

**Methods:**

Parents (*n* = 799) of 5–12-year-old children completed a validated FPP Item Bank and the Children’s Eating Behaviour Questionnaire. Latent Class Analysis (LCA) was used to identify distinct FPP profiles. Regression analyses were used to explore associations between FPP profiles, sociodemographic variables (race, sex and education) and children’s eating behaviours (emotional overeating, food responsiveness, food fussiness and satiety responsiveness).

**Results:**

LCA revealed 6 FPP profiles: *healthy eating environment*, *high engagement, reactive, high structure, controlling* and *low engagement*. Relative to their non-White counterparts, White parents were more likely to belong in the *healthy eating environment*, *high structure* and *low engagement* profiles. Relative to fathers, mothers were more likely to fall in the *healthy eating environment* compared to *low engagement* profile. Parents with some post-secondary education were more likely to belong in the *healthy eating environment*, *high structure* and *reactive* profiles compared to the *controlling* profile. Emotional overeating and food responsiveness scores were lowest for *healthy eating environment*, *high structure*, *low engagement* profiles. Parents in the *healthy eating environment* profile also reported lower food fussiness scores compared to parents in the *high engagement*, *high structure*, *reactive* and *controlling* profiles.

**Conclusions:**

Findings suggest that a continuum of 6 FPP profiles may be present among Canadian parents, representing parents who use either all (*high engagement*), some (*healthy eating environment, reactive, high structure, controlling*) or little (*low engagement*) of the FPP examined. Future longitudinal research should evaluate how various FPP profiles influence the development of children’s eating behaviors, dietary intakes and weight status.

**Supplementary Information:**

The online version contains supplementary material available at 10.1186/s12966-021-01119-6.

## Introduction

Children’s eating behaviors are influenced by a multitude of factors interacting at multiple levels including the community, household/family and child-level [1, 2]. Within the home environment, food parenting practices (FPP) (strategies and behaviours parents use to influence children’s nutrition-related behaviours and outcomes [3]) can play an integral role in children’s eating habits and weight status [4]. Vaughn and colleagues have proposed a content map presenting 3 overarching food parenting constructs (coercive control, structure and autonomy promotion) as well as specific practice subconstructs [3]. The coercive control domain includes strategies such as pressure to eat, restriction, using food as reward (or as a way to control negative emotions), all of which have been linked with emotional overeating in children [5, 6]. The structure domain includes noncoercive forms of FPP such as guided choice, routines and rules, modeling, vegetables and fruit availability and accessibility to help children maintain health-promoting behaviours [3]. Finally, the autonomy support domain is exemplified by FPP such as nutrition education, child involvement, encouragement, praise, reasoning and negotiation [3].

Examining how single FPP influence children’s eating behaviours is problematic because it ignores the co-occurrence of various FPP with one another (parents draw on a wide range of FPP which may cluster with one another [3, 7]). FPPs can also be fluid (parents may use various FPPs apparently in conflict with one another depending on a specific situation or context [8]). Within the parenting literature which recognizes that parents use multiple FPP [3, 7–10], there has been calls to use more person-centered approaches to data analysis (assigning parents to food parenting ‘profiles’ based on multiple dimensions) rather than a variable-centered approach (that ignores the interrelationships between measures) [11].

To date, limited research has examined the co-occurrence of FPP and which profile of FPP influence children’s eating behaviours. Drawing on a relatively small sample of parents (*n* = 150) with young children (5–7 years), Jennings et al. [12] identified 2 groups of parents based on varied combinations of parenting styles and practices using latent profile analysis. Parents in group 1 were more likely to use both authoritarian and permissive parenting styles in combination with coercive controlling FPP, whereas parents in group 2 reported using a more authoritative parenting style. Parents in the first group reported having children who were more food responsive and lacked internal cues for satiety compared to parents in the second group. Drawing from a larger representative sample of U.S. parent-adolescent dyads (*n* = 1657), Thomson et al. [13] identified a continuum of 5 latent classes (parent-adolescent groups) based on the use of FPP related to fruit and vegetables and found that both demographic (child age, sex) and dietary characteristics (fruit and vegetable intakes) were associated with latent class membership.

Knowledge on what clusters of FPP are used and which of these impact children’s eating behaviours can help guide clinicians design effective, person-centered interventions to guide parents towards health promoting FPPs. Therefore, the purpose of this study was to identify profiles of parents based on their use of various FPP. Secondary aims included examining sociodemographic factors associated with parent profiles and evaluating whether children’s eating behaviours differed across parent profiles.

## Methods

### Participants

This cross-sectional study drew from a sample of 799 parents of 5–12 years old children. This web-based panel of participants were recruited by Insight West, a marketing research company in British Columbia, Canada. The sampling procedure used a quota sampling approach by sex, income (using the 2015 median income of Canadian parents), and race or ethnicity (White, Asian, South Asian, and others) to ensure a diverse representation of Canadian parents. The research protocol was approved by the Research Ethics Board of the University of British Columbia, and participants provided their signed informed consent.

### Measures

#### Food parenting practices

Parents completed the online FPP Item Bank, which drew on an expert-informed conceptual framework assessing three key domains of FPP (autonomy promotion, control, and structure) and associated food parenting practices [14]. The psychometric properties of the Food Parenting Practices Item Bank has been previously validated using advanced psychometric methods [15]. Definitions and descriptive statistics for each of the 11 constructs measured in the item bank are provided in Table 1. A 1–5 response scale was used to measure each construct. Respondents’ answers were all recoded so that a higher score suggests a higher endorsement (“strongly disagree”- “strongly agree”) or higher frequency of use (from “never” to “often” or from “never” to “5 to 7 times per week”) for each construct. Constructs were dichotomized above and below the median of the original 5-point response format, where a higher score indicated more agreement or frequency in the use of a food parenting practice. We used the median to dichotomize each indicator variable in order to control for the social desirability bias inherent to self-reporting and to account for the ceiling or flooring effect with some of the constructs (see Table 1).

**Table 1:** Description of food parenting practice constructs measured in the Food Parenting Practice Item Bank [15]

Domain	Constructs	Description	n	Item Response Modeling Reliability	Mean	Median	SD	Skewness	Kurtosis
Control	Restriction for weight (4 items)	Parents keep a record of how much the child eats or talk to the child about losing weight.	595	0.79	2.1	2.0	0.9	0.9	3.5
	Coercive control (23 items)	Parents regulate children’s behaviours throughout the use of controlling and manipulative strategies.	555	0.96	2.3	2.2	0.8	0.7	3.2
Structure	Accommodate the child (5 items)	Parents allow their child to self-regulate their food intake without setting expectations.	787	0.82	2.4	2.2	0.7	0.7	3.7
	Covert control (4 items)	Parents restrict the availability and accessibility of sweet and salty treats at home.	586	0.81	2.7	2.8	1.0	0.1	2.5
	Nondirective support (8 items)	Parents model healthy behaviours and suggest their children to eat healthy without forcing them.	580	0.88	3.2	3.1	0.8	−0.1	3.0
	Redirection (2 items)	Parents negotiate with their children to limit their consumption of unhealthy food.	787	0.67	3.1	3.0	0.9	−0.3	3.1
	Provide healthy eating opportunities (9 items)	Parents systematically provide exposure to healthy foods (e.g. vegetables) at home.	592	0.87	3.7	3.7	0.7	−0.6	3.9
	Meal routines (4 items)	Parents have set routines related to food at home.	790	0.78	4.0	4.0	0.8	−0.7	3.1
	Rules and limits (9 items)	Parents have expectations about the quantity and quality of foods consumed by the child.	769	0.88	3.7	3.8	0.7	−0.5	3.3
Autonomy promotion	Child involvement (4 items)	Child is involved in meal preparation and decision-making about food.	790	0.87	3.0	3.0	0.7	0.1	3.0
	Autonomy support (14 items)	Parents promote healthy eating behaviours through education and encouragement.	567	0.93	3.1	3.1	0.8	−0.1	3.1

The validated food parenting practice item bank included 86 items measuring 11 food parenting practice constructs [15]. For each item, the questionnaire provided one of three 1–5 response scales: “never” to “often”, “never” to “5 to 7 times per week” and finally “strongly disagree” to “strongly agree”. Item Response Modeling reliability is an empirical reliability score which considers the ordinal nature of the data. For all but one construct (*Accommodate the child*), a higher score indicates higher endorsement of the construct. For example, a higher score for the construct rules and limits indicates a higher endorsement of structure-like food parenting practices while a higher score for the construct *Accommodate the child* indicates a lower endorsement of structure-like food parenting practices

#### Children’s eating behaviours

The Children’s Eating Behaviours Questionnaire [16] is a validated questionnaire designed to assess children’s appetite using 8 factors which have been divided into two 2 dimensions: food approach and food avoidance [17]. In this study, we used a reduced version of the questionnaire (20 items) to measure 4 specific behaviours: emotional overeating (4 items measuring whether the child eats more in presence of negative emotions), food responsiveness (5 items measuring whether the child wants to eat for pleasure in the absence of hunger cues), food fussiness (6 items measuring whether the child is selective with foods) and satiety responsiveness (5 items measuring whether the child gets full before his/her meal is finished). These eating behaviours were chosen as they reflect individual differences in eating self-regulation and have been linked with child weight status in previous research [18]. While emotional overeating and food responsiveness have been described as falling within the “food approach” dimension and refer to a movement towards or desire for food, food fussiness and satiety responsiveness fall within the “food avoidant” dimension and involve movement away from food [17]. These constructs were measured using a 1–5 response scale assessing the frequency of the behaviour (from “never” to “always”) in which the higher the score, the more frequent the behaviour. Constructs’ Cronbach’s α for the Children’s Eating Behaviour Questionnaire range from 0.72 to 0.91 [16].

#### Socio-demographic variables

Other variables of interest included parental socio-demographic characteristics. Demographic data included parental age, sex, marital status and race/ethnicity. The original race/ethnicity variable was adapted from the Canadian population-based surveys and posed to parents as follows: “People living in Canada come from different cultural and racial backgrounds. What is your racial or ethnic background?” with 8 possible response options: White/European, Aboriginal (e.g. North American Indian, Metis, Inuit, etc.), Chinese, South Asian (e.g. East Indian, Pakistani, Sri Lankan, etc.), Black, South East Asian (e.g. Vietnamese, Malaysian, Filipino, etc.), Japanese, Other (specify). To describe our parent sample (Table 2), we used a recoded race/ethnicity variable classifying parents as White/European vs. non-White, Asian (Chinese, Southeast Asian, or Japanese), South Asian (East Indian, Pakistani, Sri Lankan), and Other (Other, Aboriginal or Black). For objective 2 (correlates of profile membership), we dichotomized the recoded race/ethnicity variable into White vs. non-White due to the small number of participants within each of the non-White racial groups. Socio-economic data included total household income and parental educational attainment. The original parental education variable included 7 potential response options: some high school, high school degree or GED, some college or university, college or non-university certificate, Bachelor’s degree, university degree above the Bachelor’s level, or professional degree (e.g. MD, DDS, JD). To facilitate comparison with previous studies [19, 20], the educational attainment variable was dichotomised into “no post-secondary education” (i.e. high school degree or GED or below) vs. “some post-secondary education”.

**Table 2:** Characteristics of participants in the Food Parenting Study

Characteristics	% / mean ± SD
Parent age	33.1 ± 8.5
Sex, % female	50%
Marital status, % married	86%
Race/ethnicity	
White	51%
East Asian (Chinese, Japanese, Southeast Asian, etc.)	22%
South Asian (East Indian, Pakistani, Sri Lankan, etc.)	16%
Black, Aboriginal, or ‘other’	11%
Parental education^a^	
No post-secondary education	13%
Post-secondary education	87%
Household income ($CAN)	
Less than $50,000	22%
$50,000 to $99,999	51%
$100,000 to $149,999	18%
$150,000 or higher	93%
Children’s age	9.1 ± 2.4

*SD* standard deviation. N = 799 parents of children aged 5–12 years

^a^Missing data on *n* = 199 respondents. All other variables have complete (non-missing) data

### Statistical approach

LCA is a statistical technique that identifies categorical latent class variables based on observed indicator variables [21]. In the context of this study, Latent Class Analysis (LCA) was used to identify latent ‘classes’ or profiles based on their use of 11 FPP. The robust maximum likelihood estimator with the expectation-maximization algorithm was used with 1000 random starts. Recommendations from Nylund-Gibson & Young [22] and Bray et al. [23] were followed when selecting the number of classes. Models from 1 up to 7 classes were explored and different fit indexes and information criteria were computed: Bayesian Information Criterion (BIC); Sample-size Adjusted Bayesian Information Criterion (SABIC); Akaike information criterion (AIC); consistent Akaike information criterion (CAIC); and approximate weight of evidence criterion (AWE). Relative indices comparing neighboring model of classes were also evaluated. For Vuong-Lo-Mendell-Rubin likelihood ratio test (VLMR-LRT) and bootstrap likelihood ratio test (BLRT), a significant *p*-value in one model (k classes) means that such model is better than the previous one (k-1 classes). For bayes factor (BF), the higher the score, the stronger the evidence for k class model over the k + 1 class model. Third, correct model probability (cmP) was used to estimate how each model is corrected by all models considered (assuming that the correct one is among them), and higher values are desirable. Finally, relative sample sizes, interpretability and utility of the obtained classes were considered when deciding on the final number of classes. After the k class model was selected, other criteria such as entropy and average posterior probability (avePP) were considered. An entropy value above 0.8 represents a good classification of the participants into the classes, whereas the avePP > 70% represents well-differentiated classes. While the term ‘class’ is standardised terminology used when describing profiles based on observed indicator variables [24], we use the term ‘profile’ in the remainder of this study to avoiding confusion with the term ‘social class’.

Multinomial logistic regression models were used to evaluate whether sociodemographic variables (parental sex, race/ethnicity, and education) were significant predictors of parent profiles. To examine associations between children’s eating behaviours and FPP profiles, multivariable linear regression models examined differences in eating behaviour scores across latent classes while controlling for parental sex, race, educational attainment as well as children’s age and sex. Missing data were handled using case-wise deletion. Significance level was set at *p* < 0.01 to account for the multiple comparisons and maintain an adequate balance between statistical power and rate of Type I error. The LCA analyses were conducted with the Mplus software version 8.4 [25]. All other analyses were conducted in Stata version 16 [26].

## Results

Table 2 shows the sociodemographic characteristics of study participants. The sample was evenly distributed between mothers and fathers of 5–12-year-old children and the majority (86%) of parents were married. Just over half of participants (51%) self-identified as White. Twenty-two percent of parents self-identified as East Asian (Chinese, Japanese and Southeast Asians) and 16% self-identified as South Asian (East Indian, Pakistani, Sri Lankan, etc.). The remainder of the sample (11%) self-identified as either Black, Aboriginal/Metis or ‘other’ racial group. Most parents (86%) had obtained some post-secondary education and 22% of the sample had an annual household income lower than CAN$50,000.

### Profiles of food parenting practices

Using the dichotomized food parenting constructs, we fitted a series of LCA models beginning with a 1-profile model and stopping with a 7-profile model. Fit indices for these 7 models are shown in Table 3. All fit indices did not converge over a single solution and this is generally the rule rather than the exception [22]. As shown in Table 3, many of the fit indices improved with more profiles and many of the fit indices suggested that a 7-profile solution might be the best fit (SABIC, AIC, CAIC, and BF). However, for some of these indices, the improvement was minimal (CAIC) and some indices (entropy) deteriorated after 5 profiles, BIC (optimal with the 5-profile model), VLMR-LRTp (optimal with the 3- or 5-profile model) and cmP (optimal with the 4-profile model). In light of the variability in the fit indices, the interpretability of the solutions from a 3- to 7- profile model was evaluated. A 6- profile solution was retained as it was deemed to yield interpretable profiles based on the 11 FPP constructs.

**Table 3:** Model fit statistics for latent profile analyses

K	LL	BIC	SABIC	AIC	CAIC	AWE	VLMR-LRT***p***	BLRT***p***	BF	cmP	Entropy
1	− 5095.77	10,265.06	10,230.13	10,213.54	10,234.47	10,239.97	–	–	0.00	0.00	–
2	− 4812.03	9777.77	9704.74	9670.06	9713.81	9725.31	0.314	< 0.001	0.00	0.00	0.621
3	− 4626.77	9487.46	9376.31	9323.54	9390.13	9407.63	**0.005**	< 0.001	0.11	0.04	0.713
4	− 4564.90	**9443.91**	9294.66	9223.79	9313.21	9336.71	0.409	< 0.001	1.24	**0.39**	0.683
5	− 4526.95	**9448.22**	9260.87	9171.90	9284.15	9313.65	**0.029**	< 0.001	1.49	0.31	**0.737**
6	−4490.83	9456.18	9230.71	9123.66	9258.74	**9294.24**	0.353	< 0.001	4.50	0.21	0.721
7	**− 4465.76**	9486.25	**9222.68**	**9097.53**	**9255.44**	9296.94	0.096	< 0.001	**8.44**	0.05	0.709

*K* number of profiles, *LL* log-likelihood, *BIC* Bayesian information criterion, *SABIC* sample-size adjusted BIC, *AIC* Akaike information criterion, *CAIC* consistent AIC, *AWE* approximate weight of evidence criterion, *p* error probability, *VLMR-LRT* Vuong-Lo-Mendell-Rubin adjusted likelihood ratio test, *BLRT* bootstrapped likelihood ration test, *BF* Bayes factor, *cmP* correct model probability. Bolded values indicate best fit for each respective statistic

**Fig. 1:**
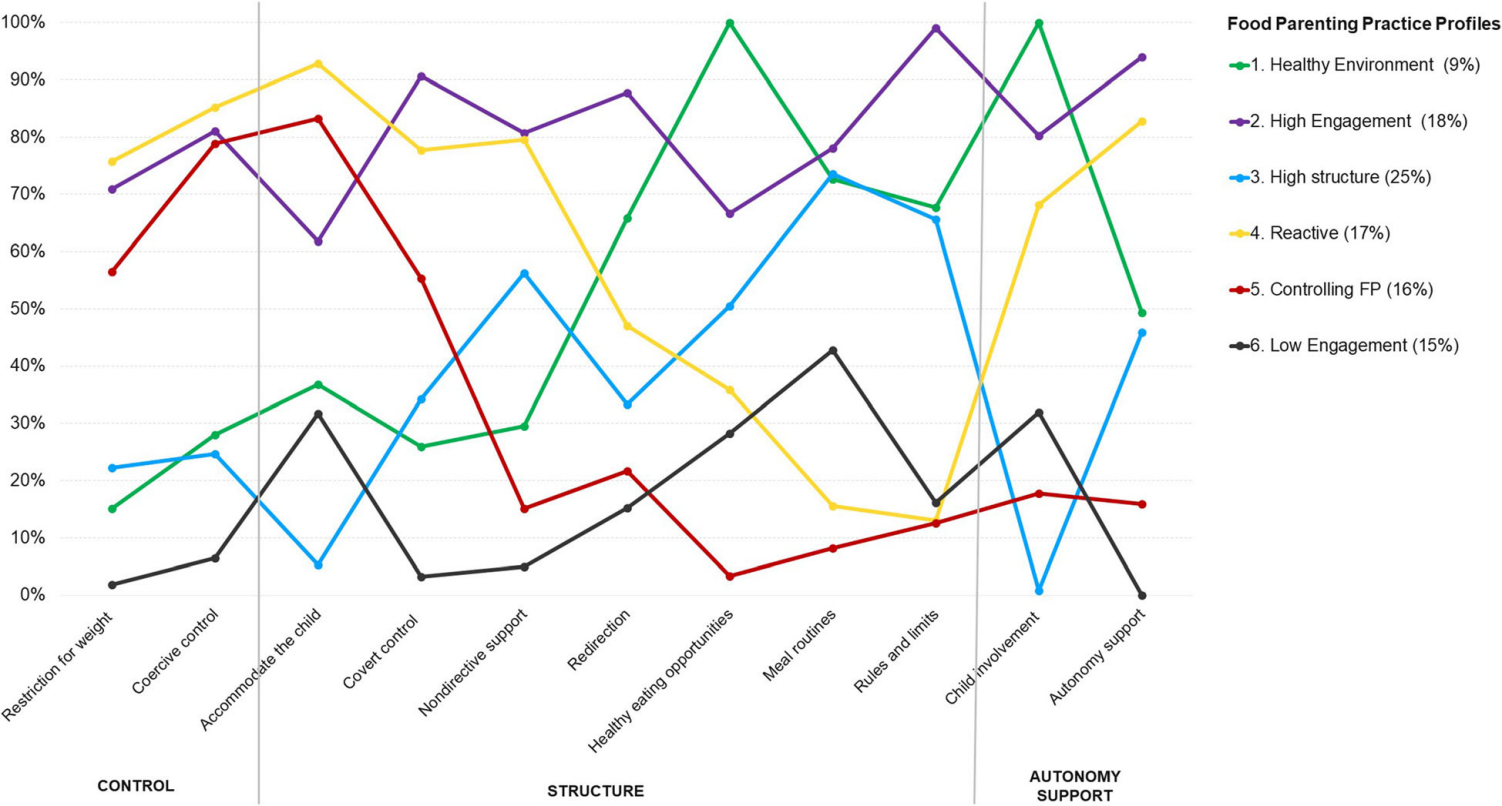
Conditional item probability plot of the 6-profile model of parental food parenting practices. The y-axis represents the probability of a “high endorsement” or “high frequency” in the use of each practice conditional on food parenting practice profile. A higher probability (%) indicate higher endorsement or frequency of use for a given construct except for the construct *Accommodate the child (*for which a lower score suggests higher structure)

Figure 1 shows the 6 parent profiles based on their probability of “low” or “high” endorsement of 11 FPP constructs. The first profile (9% of parents) was labelled *healthy eating environment* because parents in this profile showed a high endorsement towards providing healthy eating opportunities and involving their child into meal preparation and decision-making around food. The second profile labelled *high engagement* (17% of parents) included parents who showed high endorsement for almost all FPP. Parents in the *high engagement* group showed particularly high probabilities of endorsing FPP related to control and structure as well as autonomy promotion. The third profile was labelled *high structure* (25% of parents) because respondents showed high endorsement for FPP falling within the structure domain (i.e. parents tend to have a low endorsement for accommodating the child along with a high endorsement of routines and rules). *High structure* parents also reported low use of controlling FPP and lacked autonomy promoting FPPs. The fourth profile, representing 17% of the sample, was labelled *reactive* because parents in this profile showed high endorsement for controlling and autonomy promoting FPP along with a low endorsement of structure-like FPPs such as routines and rules. The fifth profile (16% of parents) was labelled *controlling* because parents showed only high endorsement of controlling FPP along with low endorsement of structure and autonomy promoting FPP. Finally, the last and sixth profile (15% of parents) was labelled *low engagement* parents because these FPP users showed low endorsement for all 11 FPP.

### Predictors of latent FPP profiles

**Fig. 2:**
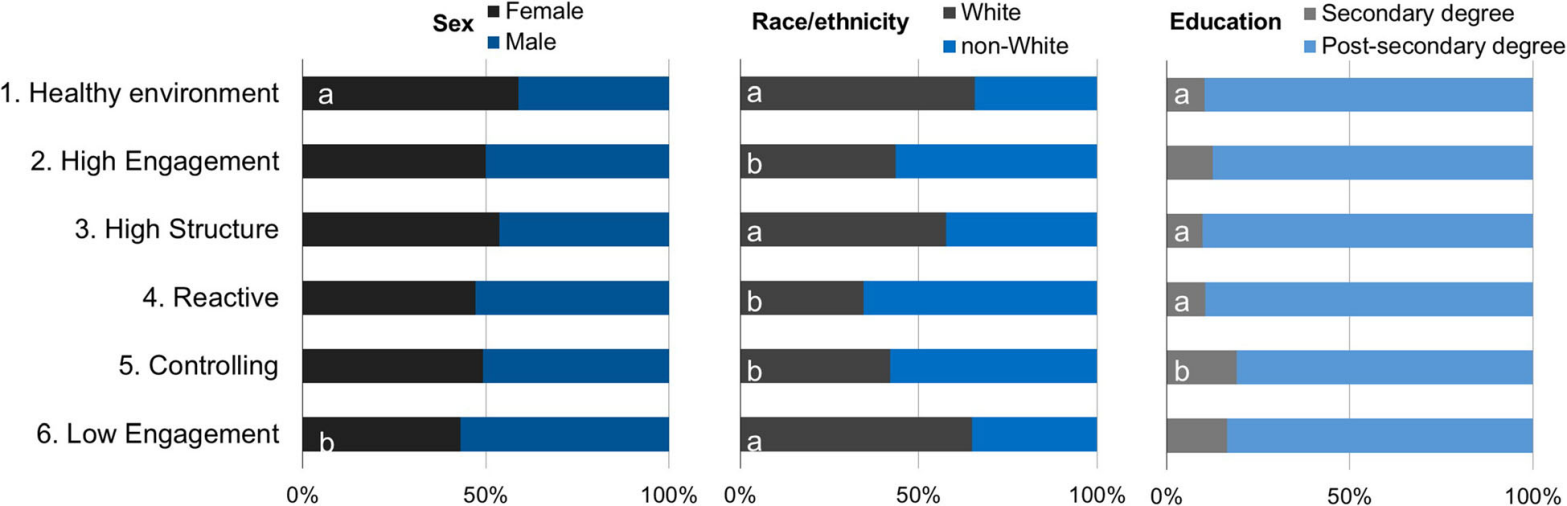
Distribution of parental characteristics across 6 food parenting practice profiles. The proportion of female/male, White/non-White, low vs. high education parents across food parenting practice profiles is different for profiles that do not share the same letter (**a**, **b**)

Figure 2 shows the sex, racial and educational attainment distribution of parents within each of the 6 parent profiles (Supplemental Table 1 shows the exact proportion or percent (%) of demographic characteristics within each parent profile). Mothers were more likely to belong to the *healthy eating environment* profile compared to the *low engagement* profile whereas a higher proportion of fathers fell in the *low engagement* profile compared to the *healthy environment* profile. Relative to their non-White counterparts, White parents were more likely to belong in the *healthy eating environment*, *high structure* and *low engagement* profiles compared to the *high engagement*, *reactive* and *controlling* profiles. Relative to parents with no post-secondary education, parents with some post-secondary education were more likely to belong in the *healthy eating environment*, *high structure* and *reactive* profiles compared to the *controlling* profile.

### Children’s eating behaviours across latent profiles

Children’s mean scores for the 4 eating behaviours examined (emotional overeating, food responsiveness, food fussiness and satiety responsiveness) across the six parent profiles are shown in Table 4. Parents in the *high engagement*, *reactive* and *controlling* profiles reported significantly higher scores for emotional overeating and food responsiveness compared to parents in the *healthy eating environment*, *high structure* and *low engagement* profiles. Parents in the *high engagement*, *high structure*, *reactive* and *controlling* profiles also reported higher food fussiness scores compared to parents in the *healthy eating environment* profile. Finally, parents falling in the *high engagement* and *reactive* profiles reported significantly higher satiety responsiveness scores compared to parents in the *healthy eating environment and low engagement* profiles. Parents in the *reactive* profile also reported higher satiety responsiveness scores compared to parents in the *high structure* profile.

**Table 4:** Average children’s eating behaviour scores across 6 food parenting practice user profiles

	Children’s eating behaviour scores			
Latent profiles	Emotional overeating	Food responsiveness	Food fussiness	Satiety responsiveness
1. Healthy eating environment	1.72 ± 0.10	2.25 ± 0.09	2.36 ± 0.09	2.67 ± 0.08
2. High engagement	2.52 ± 0.08	2.91 ± 0.08	2.79 ± 0.07	3.01 ± 0.06
3. High structure	1.70 ± 0.07	2.28 ± 0.06	2.71 ± 0.06	2.78 ± 0.05
4. Reactive	2.57 ± 0.08	2.97 ± 0.07	2.87 ± 0.07	3.11 ± 0.06
5. Controlling	2.44 ± 0.09	2.69 ± 0.08	2.97 ± 0.07	2.91 ± 0.06
6. Low engagement	1.60 ± 0.09	2.19 ± 0.08	2.68 ± 0.07	2.69 ± 0.06
Latent profile differences^a^	1, 3, 6 vs 2, 4, 5	1, 3, 6 vs. 2, 4, 5	1 vs. 2, 3, 4, 5	1, 6 vs. 2, 4 3 vs. 4

Behaviour scores can vary from 0 to 5 points where the higher the score, the more frequent the behaviour

^a^Multivariable linear regression models examined differences in CEBQ scores (emotional overeating, food fussiness, food responsiveness and satiety responsiveness) across the six profiles. Covariates included parental sex, race/ethnicity, educational attainment as well as children’s age and sex. Significance level was set at 0.01, to account for the multiple comparisons and maintain an adequate balance between statistical power and Type I error rate

## Discussion

The present study used data from a diverse sample of Canadian parents to identify distinct profiles of parents based on their uses of 11 FPP constructs. LCA revealed six FPP profiles ranging from very little use to parents who reported using almost all FPP examined (along with various combinations of controlling, structure and autonomy promoting FPP).

Based on evidence examining the influence of individual FPP on children’s nutritional outcomes [3], we ranked each profile from the most favorable to the least favorable profile in the following order: *healthy eating environment, high engagement, reactive, high structure, controlling,* and *low engagement* profiles. Parents falling in the *healthy eating environment* profile (the smallest profile including only 9% of the sample) showed high endorsement of structure and autonomy promoting FPP and contrasted with the *controlling* profile who reported using only controlling FPP. The *high structure* profile (the largest profile including 25% of the sample) included parents who showed little endorsement of controlling and autonomy promoting FPP but showed high endorsement of routines and rules. In contrast, parents in the *reactive* profile showed high endorsement of controlling and autonomy promoting FPP but lacked any structure. The latter profile could group parents who use two practices that are apparently in conflict with one another.

Few studies have used LCA to identify FPP profiles among parents. In a sample of ethnically diverse, low income U.S. parents, Jennings and colleagues [12] used latent profile analysis and identified two parent profiles based on both their parenting styles and FPP. Parents in the first profile included parents who used both authoritarian and permissive parenting styles along with controlling FPP (e.g. restriction, pressure to eat) (overall, a less favorable profile) whereas parents in the second profile used more an authoritative parenting style along with less controlling FPP (overall, a more favorable pattern). In contrast to Jennings et al.’s study, our study did not include parenting styles and drew on a larger sample of parents with older children. We also included a wider range of FPP as indicator variables. Our findings suggest a continuum of 6 FPP profiles may be present, representing parents who use either all (*high engagement*), some (*healthy eating environment, reactive, high structure, controlling*) or little (*low engagement*) of the FPPs examined. In another U.S. study conducted among parent-adolescent dyads, Thompson et al. identified 5 parent profiles based on parental use of pressure to eat, monitoring, modeling, encouragement, availability and child involvement regarding their child’s fruit and vegetable intake. Two of the FPP profiles were somewhat analogous to our study. The *indifferent influencers* profile reported low use of all FPPs similar to the *low engagement* profile of parent in our study; with low prevalence in both studies (14 and 15%, respectively). The *complete influencers* reported high use of all practices like the *high engagement* parents in our study. Finally, O’Connor et al. identified 3 clusters of FPPs related to vegetable and fruit consumption among U.S. parents: *indiscriminate food parenting*, *non-directive food parenting*, and *low-involved food parenting* (similar to our *low involvement* group in our study) [7]. The *non-directive food parenting* profile appears similar to the *healthy eating environment* parents by using enhanced availability and teachable moments’ practices, but less firm discipline practices than the other clusters. Taken together, our findings suggest that there are parent profiles who use either multiple FPP simultaneously or only a few practices to influence their children’s eating behaviours.

This study found that race/ethnicity could potentially be associated with differences in FPP profiles. However, since non-White parents in this sample constituted a heterogenous groups of parents, interpreting our findings related to potential racial or ethnic differences is limited. Previous research suggests there may be cultural differences in feeding norms and practices as well as economic disparities across racial groups which could contribute to a parent’s decision to utilize specific FPP [19, 20, 27–32]. A study conducted in a large population-based sample of U.S. parents of varied and diverse minorities groups suggest that the use of controlling FPP, such as pressuring children to eat and restricting children’s intake, is common among racial minority parents [19]. Findings from cross-sectional studies suggests that non-White Hispanics [20, 27] and Chinese-American parents [29] are more likely to endorse controlling feeding behaviours such as pressure to eat and restrictive feeding practices compared to White parents. Our findings suggest a more nuanced picture; non-White parents in our sample tended to belong to the *high engagement*, *reactive* and *controlling* profiles compared to the *healthy environment*, *high structure* and *low engagement* profiles. This suggest that while some non-White parents may use controlling practices, they simultaneously could be using autonomy promoting (*reactive* parents) as well as autonomy supportive practices (*high engagement* parents). These findings highlight the need for clinicians to consider the plurality of practices parents may use and the need for culturally competent and safe approaches to encourage health promoting FPPs among parents.

Although some research suggest that fathers employ unique sets of FPP (that set them apart from mothers) [33, 34], we found that gender was not a strong predictor of parent profiles. Compared to fathers, mothers were more likely to fall in the *healthy eating environment* profile compared to *low engagement* profile. This supports the finding by Davison et al. [34] that while fathers consider themselves responsible for feeding children, their involvement still lags behind or tends to be at a lower level than for mothers. Studies in Canada still consistently demonstrate that women remain responsible for the bulk of house work (despite men’s increasing involvement in household-related tasks), including assuming responsibility for the health and well-being of family members [35, 36].

Differences in children’s eating behaviours were observed among parent profiles, which lend support for the validity and utility of the profiles obtained with the LCA. As expected, parents who used more controlling FPP (*high engagement*, *reactive* and *controlling* parents) also reported higher emotional overeating and food responsiveness (more “food approach” behaviours). This finding is in line with previous research suggesting that parents who perceive their child as wanting to eat for emotional reasons or simply as “having a big appetite” may be more likely to employ more coercive controlling practices (e.g. restricting for weight) in order to shape their child’s dietary intakes (despite evidence suggesting these are counterproductive measures and linked with excessive weight gain [37, 38]). At the same time, we found that parents who used simultaneously controlling and autonomy promoting practices (*high engagement*, *reactive* parent profiles) also reported the highest satiety responsiveness scores (a more “food avoidant” behaviour). Previous literature suggests that using other controlling practices such as pressuring the child to eat can result in a counterproductive effect of more food avoidant behavior [39], which is reflected in our findings with satiety responsiveness. There is growing consensus of the bidirectional relationship between parental FPP and children’s eating behaviours, wherein parents may increase their controlling behaviours as a result of a concern that their child is leaving too much food on their plate [4, 40]. The finding that parents who used the most favourable combination of FPP (*healthy eating environment*) also reported the lowest food fussiness scores was not surprising and aligns with previous research indicating that structure and autonomy promoting FPP are associated with more healthful eating behaviours among children [3, 41].

Strengths of this study include a sampling approach to target mothers and fathers, a relatively large and racially diverse sample of Canadian parents, as well as the use of a validated food items bank to measure FPP. There are also important limitations that deserve consideration. This study employed a cross sectional design so no causality can be inferred between FPP profiles and children’s eating behaviours. This study assessed FPPs in a sample of elementary school-age children (age 5–12 years) and as such, it is possible that the relationships reported here may not be generalizable to younger children or adolescents. Finally, while the proportion of non-White parents in this sample (49%) was relatively high (approximately 30% of total population in Canada is non-White [42]), the analysis dichotomized the race variable into two groups (White vs. non-White parents) in order to gain statistical power when exploring racial differences among FPP profiles. In doing so, this analysis could have missed important differences in FPP profiles among non-White parents in this sample (for e.g. differences FPP profiles between East vs. South Asian parents). Future FPP research should consider focusing on visible minority parents in Canada to determine whether the profiles identified here remain relevant and are associated with eating behaviours among children.

## Conclusion

In summary, this study identified 6 profiles of FPPs among Canadian parents. Researchers and practitioners should consider that parents may use simultaneously a wide variety of FPP and therefore adopt a person-centered approach in designing interventions to encourage specific combinations of FPP associated with more healthful dietary behaviours. This study also demonstrates how parent profiles predicted children’s eating behaviours, suggesting it may be important for clinicians to provide guidance to parents to establish and maintain structure and autonomy promoting FPPs, both of which were associated with lower food responsiveness and emotional overeating. Future longitudinal research is needed to evaluate the influence of FPP parent profiles on the development of children’s eating behaviors, dietary intakes and weight status.

## Supplementary Information

**Additional file 1.**

## Data Availability

Please contact the corresponding author (LCM at lmasse@bcchr.ubc.ca) for any questions about the study including data requests or study materials.

## Abbreviations

avePP: Average Posterior Probability
AWE: Approximate weight of evidence criterion
BF: Bayes factor
BLRT: Bootstrap likelihood ratio test
BIC: Bayesian information criterion
CAIC: Consistent Akaike Information Criterion
cmP: Correct model probability
FFP: Food parenting practices
LCA: Latent class analysis
SABIC: Sample-size Adjusted Bayesian Information Criterion
VLMR-LRT: Vuong-Lo-Mendell-Rubin Likelihood Ratio Test
