# Supplementary material for: Identifying and predicting food parenting practice profiles among Canadian parents
Source: Int J Behav Nutr Phys Act. 2021 May 4;18:59. doi: 10.1186/s12966-021-01119-6 (PMC8097990; doi:10.1186/s12966-021-01119-6)
Supplement: Supplementary file 1 — Additional file 1. [file 12966_2021_1119_MOESM1_ESM.docx]

**Supplemental Table 1.** Distribution of parental characteristics across the six food parenting practice profiles

|  | **Latent Class** | | | | | |  |
| --- | --- | --- | --- | --- | --- | --- | --- |
|  | **1** | **2** | **3** | **4** | **5** | **6** | **Group differences^1^** |
| **Parent characteristics, %** | **Healthy eating environment** | **High Engagement** | **High Structure** | **Reactive** | **Controlling** | **Low Engagement** |  |
| Female | 58.9% | 50.0% | 53.7% | 47.1% | 49.2% | 43.1% | 1 *vs.* 6 |
| White | 65.8% | 43.6% | 57.7% | 34.6% | 42.1% | 65.0% | 1, 3, 6 *vs.* 2, 4, 5 |
| Post-secondary education | 89.6% | 87.4% | 90.1% | 89.5% | 80.9% | 83.5% | 5 *vs.* 1, 3, 4 |

^1^Group differences across latent class groups were assessed using multinomial logistic regressions of the Three-Step Method [1]. Differences across groups are significant at p-value<0.01.

Reference

1. Vermunt JK. Latent class modeling with covariates: two improved three-step approaches. Polit Anal. 2010;18:450–69.
